# The dynamic Alp landscape of *Streptococcus dysgalactiae*

**DOI:** 10.3389/fcimb.2026.1770747

**Published:** 2026-04-10

**Authors:** Camilla Olaisen, Alba Kaci, Christina Gabrielsen Ås, Jan Egil Afset, Oddvar Oppegaard

**Affiliations:** 1 Department of Medical Microbiology, St. Olavs Hospital, Trondheim, Norway; 2 Clinic of Laboratory Medicine, St. Olavs Hospital, Trondheim, Norway; 3 Center for Laboratory Medicine, Østfold Hospital Trust, Grålum, Norway; 4 Department of Clinical and Molecular Medicine, Faculty of Medicine and Health Sciences, Norwegian University of Science and Technology (NTNU), Trondheim, Norway; 5 Department of Clinical Science, Faculty of Medicine, University of Bergen, Bergen, Norway; 6 Department of Medicine, Haukeland University Hospital, Bergen, Norway

**Keywords:** allelic variants, interspecies gene transfer, MGE, N-terminal immunodomain, SDSD, SDSE, whole genome sequencing, alpha-like proteins

## Abstract

**Background:**

*Streptococcus dysgalactiae* is an important pathogen in both humans and animals. Alpha-like proteins (Alps) are surface-anchored adhesins and contribute to host tissue invasion. While Alps are nearly ubiquitous in *Streptococcus agalactiae* and are recognized as major virulence factors in *Streptococcus pyogenes*, their presence and diversity in *S. dysgalactiae* remain poorly characterized.

**Methods:**

We screened 1,243 *S. dysgalactiae* genomes of human and animal origin from Norway, Japan, Canada, and Australia for presence of *alp* genes. A subset of 32 strains from Norway was further sequenced using long-read technology (Nanopore) to resolve tandem repeat regions of *alp* genes.

**Results:**

By screening global strain collections, we found five novel *alp* genes, designated here as *dysalp2–5* and *alp1–3 hybrid*. The *alp* genes exhibited extensive genetic heterogeneity, predominantly arising from mosaicism and tandem repeat variations. Overall, *alp* genes were detected in 18% of human and 10-15% of animal strains. There were geographic differences in prevalence and diversity of *alp* genes, yet the overall distribution did not follow a strictly clonal pattern. The *alp* genes in *S. dysgalactiae* resided on mobile genetic elements, and we detected similar elements also in other streptococcal species.

**Conclusion:**

We reveal that Alpha-like proteins are common and widely distributed among *S. dysgalactiae*, and exhibit extensive genetic heterogeneity. Our findings expand our understanding of Alp diversity and shed light on the evolutionary mechanisms that drive cross-species exchange of *alp* genes.

## 1 Introduction

*Streptococcus dysgalactiae* (SD) is a potent pathogen, capable of causing severe infections in a wide range of hosts species. SD is divided into the subspecies *dysgalactiae* (SDSD), mainly associated with bovine infections, and subspecies *equisimilis* (SDSE), comprising several distinct ecovars targeting humans, horses, dogs, swine and fish, respectively (Porcellato et al., 2021). SD is phylogenetically closely related to *Streptococcus pyogenes* (group A streptococcus, GAS) and *Streptococcus agalactiae* (group B streptococcus, GBS), sharing overlapping clinical manifestations, genetic features, and virulence determinants (Franken et al., 2001; Rosinski-Chupin et al., 2019; Xie et al., 2024). Moreover, horizontal genetic exchange occurs between these closely related species, including transfer of bacteriophages and integrative conjugative elements (ICEs) (Giovanetti et al., 2014; Smyth et al., 2014).

**Figure 1:**
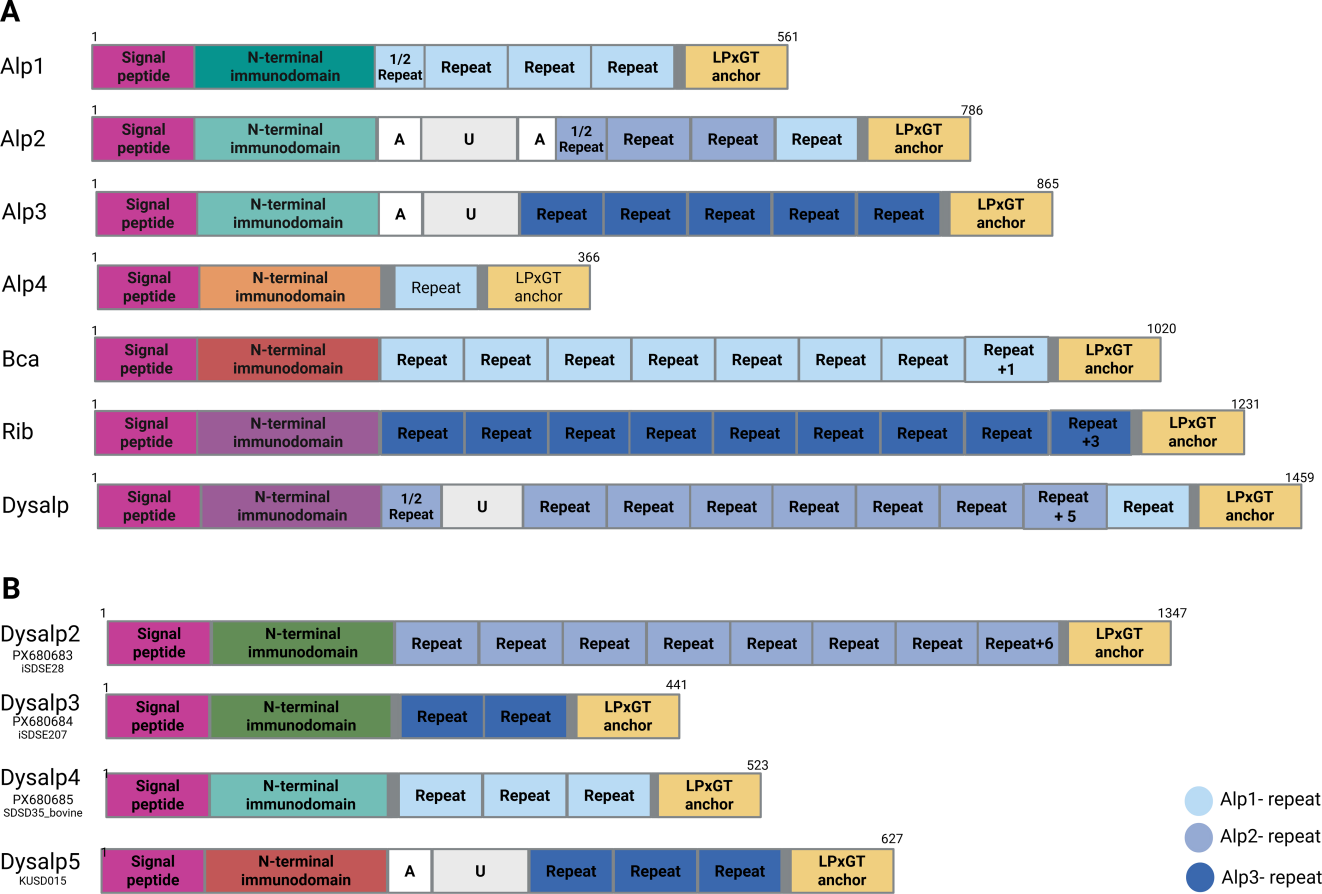
Schematic structure of Alpha-like proteins (Alps). The Alps are composed of a signal peptide and an N-terminal immunodomain followed by a region of variable number of tandem repeats, and a C-terminal transmembrane region including the cell wall anchor region with an LPXTG motif. A- and U- regions are also present in some proteins. The seven previously characterized Alps **(A)** comprise one of five different N-terminal immunodomains and one or two of the three different tandem repeat sequences. **(B)** Novel Alps described in this study.

Alpha-like proteins (Alps) are a group of surface-localized virulence factors that are shared by GAS, GBS and SD. The Alp protein family are best characterized in GBS, and includes Alpha C (*bca*), Alp1 (*alp1*), Alp2 (*alp2*), Alp3 (*alp3*), Alp4 (*alp4*), and Rib (*rib*) (Maeland et al., 2015). An Alp3 homolog, R28, has been documented in GAS (Stålhammar-Carlemalm et al., 1999), and in 2007 Dysalp (*dysalp*) was described in SD (Creti et al., 2007). The members of the Alp protein family are chimeras with a mosaic structure composed of a signal peptide and an N-terminal immunodomain of 220–230 amino acids (aa), followed by a variable number of identical tandem repeats, each comprising approximately 80 aa, and a C-terminal transmembrane region (∼44 aa long), including the cell wall anchor region with an LPXTG motif (Lachenauer et al., 2000; Creti et al., 2007). In addition, some Alps harbor U- and A-regions, non-tandem repeats of ∼137 aa and ∼51 aa, respectively (Lachenauer et al., 2000) (**Figure 1A**).

The Alps are believed to enhance bacterial virulence by functioning as adhesins. Alpha C has been found to mediate invasion of vaginal epithelial cells through binding to glycosaminoglycan (Baron et al., 2004). In GAS the mobile genetic element (MGE) ICE6180-RD2 carrying the *alp3* homologue *R28*, has been shown to promote adhesion to human epithelial cells *in vitro* and enhance vaginal colonization in a murine model (Stålhammar-Carlemalm et al., 1999; Jain et al., 2019; Eraso et al., 2020; Roshika et al., 2021). Whereas nearly all clinical strains of GBS carry an *alp* gene (Gabrielsen et al., 2017; McGee et al., 2021), *R28* seem to be restricted to serotype-specific lineages of GAS. Notably the gene is present in all serotype M28 strains, which are strongly associated with puerperal sepsis and neonatal infections (Green et al., 2005). Recently, Roshika et al. (2021) demonstrated that transferring the M28-associated ICE6180-RD2 element to other GAS serotypes increased the recipient isolates’ vaginal colonization rates in a murine model. Interspecies dissemination of *alp* genes through ICEs has been postulated but not experimentally verified.

PCR tiling has suggested that *dysalp* in SD is situated on an ICE similar to ICE6180-RD2 (Sitkiewicz et al., 2011), but this has not been verified by whole genome sequencing. Moreover, the prevalence and diversity of Alps in SD have not been extensively explored.

Using whole genome sequences of clinically relevant SD strains previously isolated and sequenced from various host species in Norway, along with sequences from Australia, Japan, and Canada, we investigated the occurrence and diversity of *alp* genes. The aim was to gain insight into the global distribution and diversity of the Alp protein family in SD.

## 2 Materials and methods

### 2.1 Bacterial genomes included in the study

SD strains of both human and animal origin from previous surveillance studies conducted in Norway were included (Kaci et al., 2023; Oppegaard et al., 2023; Glambek et al., 2024). Briefly, this comprised all strains (n=274) causing human bloodstream infections in Norway in 2018 (Kaci et al., 2023), and all human blood culture strains (n=277) collected in Bergen, western Norway, during 1999-2017 (Oppegaard et al., 2023). In addition, we included SD strains from bovine/ovine sources (n=85) associated with mastitis and collected by TINE SA mastitis laboratory (Molde, Norway) in 2018, as well as clinical strains from other animals (n=52) submitted to the Norwegian Veterinary Institute in 2018-2019 (Glambek et al., 2024). Primary laboratories performed species identification as described previously (Kaci et al., 2023). The vast majority of the included SD strains have been previously sequenced, but in the present study we sequenced the remaining 141 human-associated SD strains from Bergen, and two animal associated SD strains from the Norwegian Veterinary Institute.

A further 562 previously published SD genomes of human origin from Canada (n=122, collected in 2012-2014) (Lother et al., 2017), Australia (n=294, collected in 2003-2005) (Xie et al., 2024), and Japan (n=146, collected in 2005-2021) (Shinohara et al., 2023) were downloaded from GenBank and included in this study.

### 2.2 Whole genome sequencing

Bacterial culturing and DNA extraction was performed as previously described (Oppegaard et al., 2017). Whole genome sequencing of the majority of SD isolates of human origin (n=396), as well as all SD from animal origin were sequenced using the Illumina DNA Prep (Illumina) and a NovaSeq 6000 system (Illumina) to produce 2x 150bp paired end reads. The remaining 155 SD isolates of human origin (part of the NORM 2018 material) were sequenced at Østfold Hospital Trust on an Ion S5XL system (Thermo Fisher Scientific) as previously described (Kaci et al., 2023).

To overcome limitations of short-read sequencing, including fragmented assemblies and unresolved tandem repeats in Alp genes, a subset of 32 SD strains of both human and animal origin, previously sequenced by Illumina or Ion Torrent technology, were additionally sequenced by Oxford Nanopore Technology. Genomic DNA was prepared by dissolving bacterial colonies in 200 μL TE buffer containing 1.5 mg/mL lysozyme, 0.5 mg/mL proteinase K (Qiagen) and 250 U/mL mutanolysin (Merck), prior to 15 min incubation at 37 °C, 15 min at 65 °C, and addition of 100 mg/mL RNaseA. Genomic DNA was subsequently extracted using the EZ1 DNA Tissue kit (Qiagen) with an EZ1 Advanced XL extractor (Qiagen). Sequencing libraries were prepared using the rapid sequencing kit SQK-RBK114.24 followed by sequencing on a FLO-MIN114 flow cell (R10.4.1) using a MinION Mk1b instrument (Oxford Nanopore Technologies).

### 2.3 Quality control and *de novo* assembly

Short-read sequencing data were initially preprocessed using Trimmomatic v0.39 (Bolger et al., 2014) (for the Illumina HiSeq system) or by other incorporated software plug-ins (for the Ion Torrent system), to remove adapters, quality trim and filter prior to *de novo* assembly by Spades v5.14 (Bankevich et al., 2012).

Long-read sequencing data were processed using an in-house pipeline for basecalling and demultiplexing with Dorado 0.4.2, assembly using Flye 2.9.2 and polishing by Racon 1.5.0, Medaka 1.1.1 and homopolish 0.3.4.

Prior to annotation, the assembly metrics of all assembled contigs/genomes were evaluated by QUAST v.5.2.0 (Gurevich et al., 2013) and genome completeness and quality were further evaluated using CheckM2 v.1.1.0 (Chklovski et al., 2023). Genomes failing the quality criteria (contig number <150, completeness >95% and contamination <5%) were excluded from downstream analyses.

### 2.4 Annotation and phylogeny

All assembled genomes were annotated by RAST v1.073 (Aziz et al., 2008) and genotyped using the multilocus sequence typing web-tool provided by the Center for Genomic Epidemiology (Larsen et al., 2012). Double locus variants of multi locus sequence types were clustered into clonal complexes using Phyloviz (Nascimento et al., 2017). Single-nucleotide polymorphisms were called using Snippy with default settings and the SDSE type strain NCTC13762 as reference (Seeman, 2015). A maximum likelihood phylogeny was then generated using IQ-TREE and annotated using the Interactive Tree of Life (Letunic and Bork, 2007). In accordance with the phenotypic definition proposed by Vieira et al. (1998), we defined SDSD *in silico* as genomes harboring the Lancefield group C-antigen operon, lacking the streptolysin S operon (corresponding to an α- or nonhemolytic reaction on blood agar), and lacking the streptokinase gene (inferring that streptokinase activity on human plasminogen does not occur). All other genomes were classified as SDSE. Genomes downloaded from GenBank (PRJDB12179, PRJNA325743, and PRJEB35476) were *de novo* assembled (SPAdes) and annotated (RAST) as described above.

### 2.5 Identification of *alps* in SD genomes

Genomes were screened for the presence of *alp* genes *bca* (M97256), *rib* (U58333), *alp1* (AH013348), *alp2* (AF208158), *alp3* (AF245663), *alp4* (AJ488912), and *dysalp* (AH015632) using the BLASTn algorithm, or Annotation & Predict tab/tool and a customized database consisting of the known sequences of the *alp* genes, in Geneious Prime v.2025.1.3. Identified *alp* genes encoding N-domains and/or repeat regions with <80% protein sequence similarity to established Alp proteins (Alpha C, Rib, Alp1, Alp2, Alp3, Alp4, and Dysalp) were classified as new members of the Alp protein family. Moreover, *alp* genes displaying novel combinations of N-domain and repeat regions were also considered novel *alp* variants. The genomes of SD strains included in this study were further screened for integrative conjugative elements (ICEs) using ICEscreen, and potential ICEs in the *alp*-region were verified by manual inspection and BLAST-search (Lao et al., 2022).

### 2.6 Detection of *alps* in global genome repositories

Detection of selected proteins in global genome repositories was performed by protein blast (using blast+ with evalue 0.05, best_hit_overhang 0.25 and best_hit_score_edge 0.1) of a fragment containing the N-terminal immunodomain and the first repeat domain against the full non-redundant protein BLAST database (downloaded 14.04.2025). Results were filtered based on alignment length of ≥ 90%.

### 2.7 3D protein modelling

Protein modelling of selected protein sequences was performed using the AlphaFold server (Abramson et al., 2024), with the AlphaFold 3 model. CIF files of the highest overall pTM quality scores were converted to pdb-format using GEMMI tools in WebAssembly (Gemmi, 2024).

## 3 Results

We explored a global collection of 1,250 SD genomes, comprising 1,113 and 137 strains from human and animal origin, respectively. Seven genomes were of insufficient quality and were excluded from further analyses. The characteristics of the 1,243 included genomes are presented in **Supplementary Table 1**. Based on *in silico* predicted species delineation, 1,159 and 84 were characterized as SDSE and SDSD, respectively.

### 3.1 Novel members of the Alp protein family

Whole genome sequences of all the included bacterial strains were screened for members of the Alp protein family genes, revealing substantial allelic diversity and several novel *alp* variants (**Figure 1B**). In eight genomes we observed *alp* genes encoding an N-terminal immunodomain with only ∼70% identity to previously described N-terminal immunodomain sequences. This novel N-terminal immunodomain was in some strains followed by tandem repeat regions highly similar to those described in Alp2, whereas in others it was combined with Alp3 tandem repeat regions. We designated these two new variants as Dysalp2 (*dysalp2*) and Dysalp3 (*dysalp3*), respectively (**Figure 1B**). We also observed Alps with novel combinations of canonical N-terminal immunodomains and repeat regions. A few SDSD strains harbored *alp* genes composed of an Alp2/Alp3 N-terminal immunodomain and Alp1/Alp4/Alpha C repeat regions, herein denoted as Dysalp4 (*dysalp4)*. Moreover, we observed *alp* genes combining the N-terminal immunodomain of Alpha C and the repeat-region from Alp3. This allelic variant was designated Dysalp5 (*dysalp5*).

### 3.2 Mosaicism of *S. dysgalactiae alp* genes

**Figure 2:**
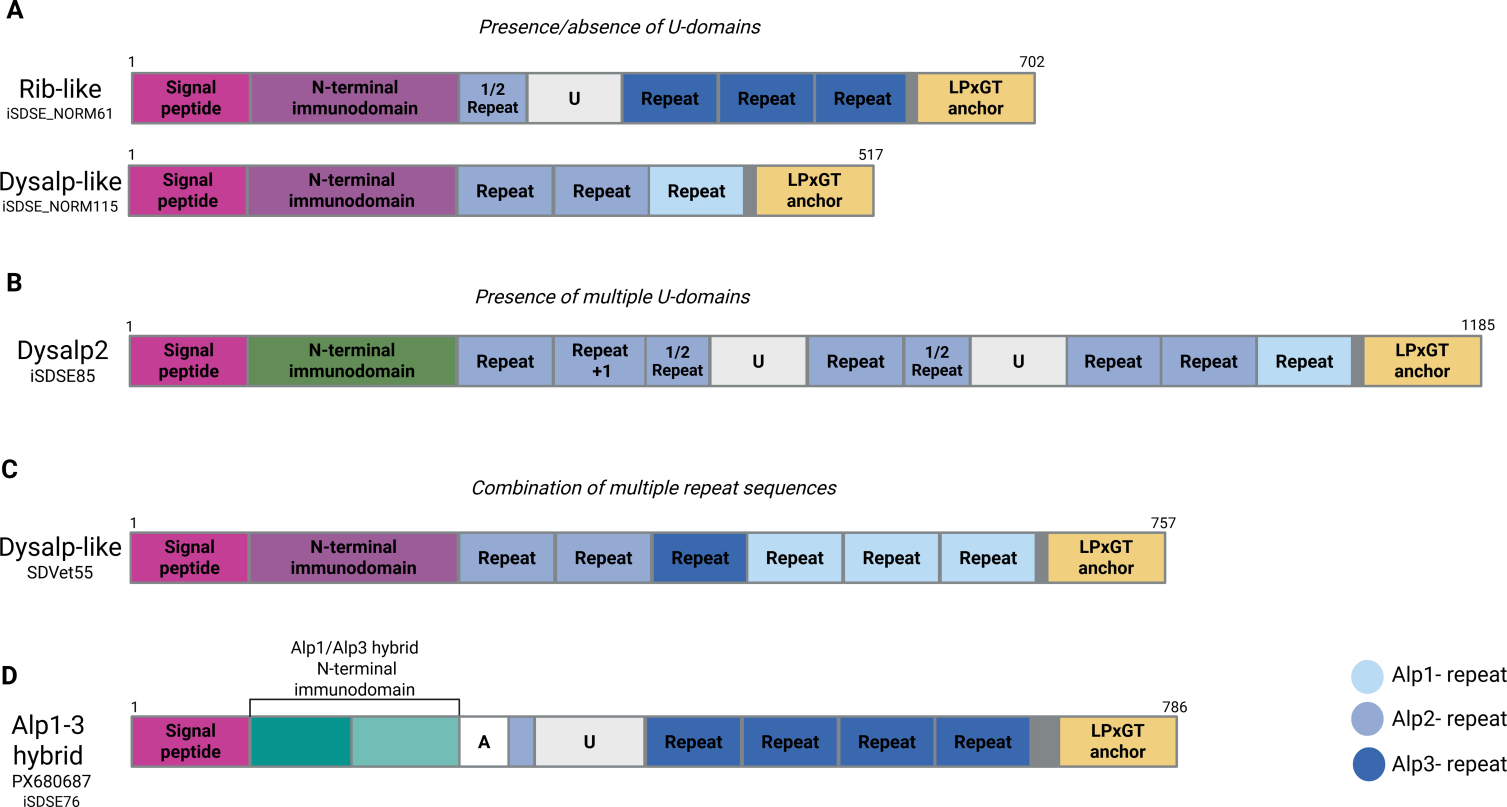
Mosaicism of SD Alp protein family genes. Mosaic variants of Alps are characterized by presence/absence of U-domains **(A)**, presence of multiple U-domains **(B)**, and combination of multiple repeat sequences **(C)**. The Alp1-3 hybrid protein harboured an N-terminal immunodomain that was a mosaic of the Alp1 and Alp2/3 immunodomains **(D)**.

Exploring the different *alp* genes identified in the SD strains, we revealed several examples of structural rearrangements and gain or loss of domains contributing to allelic diversity (**Figure 2**). In general, mosaic variants characterized by the presence/absence of U-domains, and/or combination of multiple types and numbers of tandem repeat sequences, were not classified as new *alp* family members but designated as *alp*-like in this study. For instance, several strains harbored genes encoding Dysalp proteins lacking a U-domain (Dysalp-like), or Rib proteins containing an extra U-domain ahead of the tandem repeat region (Rib-like). Moreover, some SDSE strains of human origin were characterized by the presence of multiple U-domains (**Figure 2B**).

We also observed that tandem repeat sequences varied between strains, both in type and number. Four horse-associated SDSE strains had *alp* genes encoding three different canonical repeat-variants, i.e. *dysalp*/*alp2*-repeats, *alp3*-repeats and *alp1*-repeats (**Figure 2C**). We decided to conform to naming Alp-proteins based on the combination of the N-terminal immunodomain and the first repeat segment, thus designating this as mosaic variants of Dysalp.

Interestingly, some genomes encoded an N-terminal immunodomain that was a hybrid of the N-terminal immunodomain of *alp1* and *alp2/3* (**Figure 2D**). Protein sequence alignment of this hybrid N-terminal immunodomain showed a 99% similarity to the first 87 aa in Alp1, whereas the rest of the sequence was identical to the Alp2/3 N-terminal immunodomain (100% sequence similarity). After the N-terminal region, the sequences of these strains consisted of a U-domain and tandem repeat sequences identical to Alp3. We designated this mosaic variant Alp1–3 hybrid.

Taken together, this demonstrates that *alp* genes exhibit extensive genetic rearrangement and recombination, and this mosaicism further complicates the classification of the different *alp* genes in *Streptococcal* species.

### 3.3 Prevalence of *alp* genes among *S. dysgalactiae* strains

In total, *alp* genes were detected in 199 (18%) human-associated SDSE strains, and in 26 (50%) SDSE strains and nine (10.6%) SDSD strains derived from companion animals and/or livestock (**Table 1**). The predominant *alp* gene among the human-associated strains in Norway was *alp3* in 79 (14.3%) strains, followed by *dysalp* with 19 (3.4%), and *rib* with eight (1.5%) strains. Notably, *alp3* was exclusively detected in human-associated strains, whereas *rib* and *dysalp* were also common among the animal-derived strains. The newly identified *dysalp4* was predominantly present among the SDSD strains of bovine/ovine sources (seven strains, 8.2%) and not detected among the human-associated SDSE strains.

**Table 1:** Distribution of Alp protein family genes among SD strains included in this study.

Country	Isolate type	*n*	Source	*alp1*	*alp3*	*bca*	*rib*	*dysalp*	*dysalp2*	*dysalp3*	*dysalp4*	*dysalp5*	*alp1–3 hybrid*	unspecified*	Total	*% alp*
Norway	Human	551^a)^	^c),d) and e)^	1	79	2	8	19	3	3			3	2	120	22
Norway	Various animals	52^a)^	^c) and f)^				18	5	2		1				26	50
Norway	Bovine, Ovine	83^b)^	^f)^	1							7			1	9	11
Canada	Human	121^a)^	^g)^		8										8	7
Australia	Human	293^a)^	^h)^					45							45	15
Japan	Human	142^a)^	^i)^			7		8				8	2		25	18
Total		1242		2	87	9	26	77	5	3	8	8	5	3	233	19

* Unable to assign a specific *alp* gene because the sequence was truncated.

^a)^ SDSE

^b)^ SDSD

^c)^ This study

^d)^ Kaci et al., 2023

^e)^ Oppegaard et al., 2023

^f)^ Glambek et al., 2024

^g)^ Lother et al., 2017

^h)^ Xie et al., 2024

^i)^ Shinohara et al., 2023

Several geographic differences were noted. The *alp3* gene was the most prevalent in the human-associated strain collections from Norway and Canada but was not present in the Japanese and Australian collections. Conversely, *dysalp* was frequent in the Australian (15%) and Japanese (5.8%) collections, whereas no strains harboring a *dysalp* gene were recovered from the Canadian collection. Japanese strains seemed to have a more diverging profile of *alp* genes among its strains, characterized by the presence of strains with the *alp1–3 hybrid* mosaic variant, as well as the newly identified member of *alp* genes, *dysalp5*.

**Figure 3:**
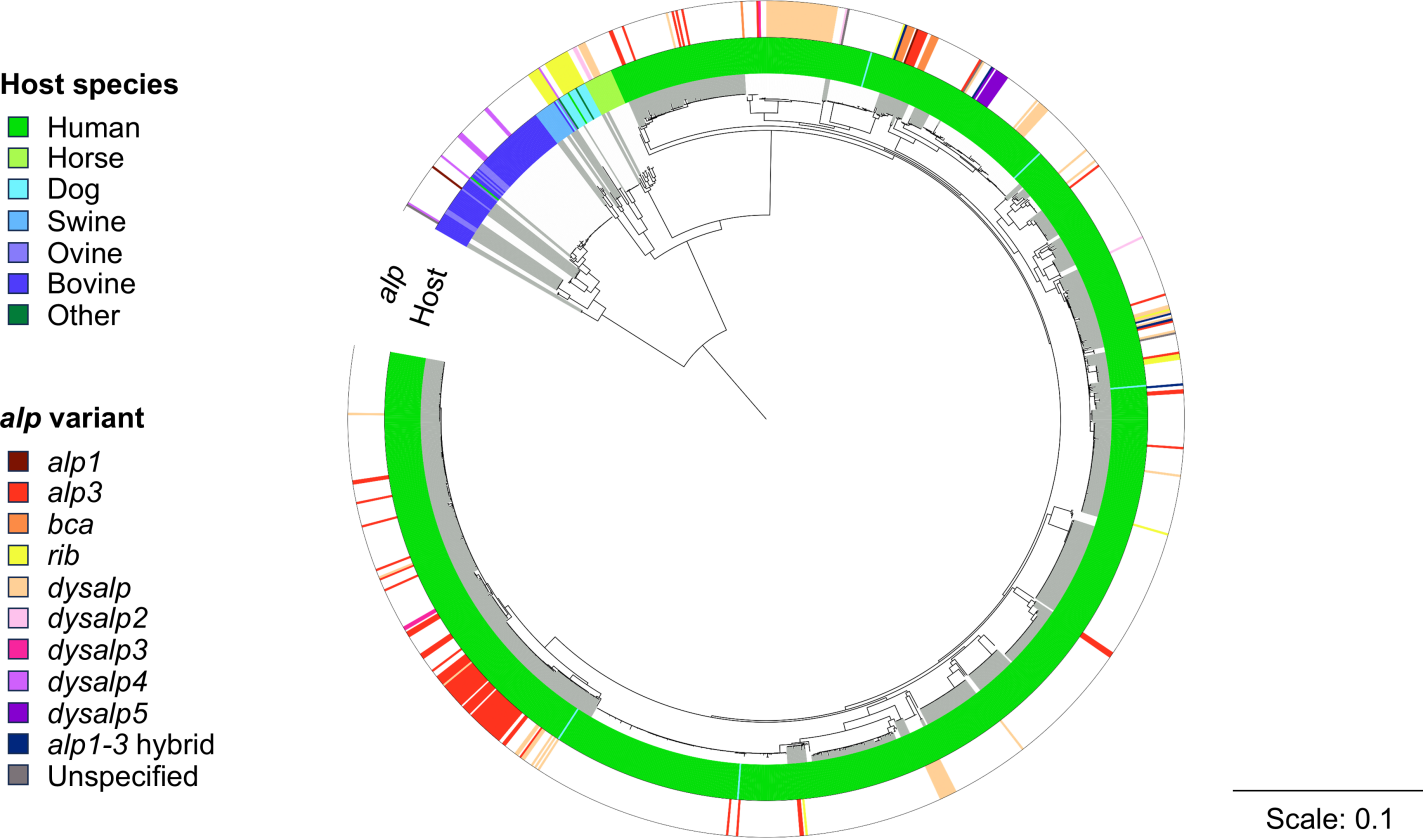
Phylogenetic analysis of SD genomes of human and animal origin included in this study. The tree is based on whole genome sequencing of the isolates. Clonal complexes are indicated by alternating background colors (white and gray). Scale indicates substitutions per site.

Phylogenetic analysis of the Norwegian strains demonstrated that the SD strains mainly grouped together based on the host-species (**Figure 3**). Although there were a few phylogenetic clusters that appeared to be strongly associated with the presence of specific *alp* genes, in general the *alp* genes did not display a clonal pattern. Nevertheless, a correlation between host species and certain *alp* genes was evident, as e.g. *alp3* was only present in SD from human origin, whereas *dysalp4* was only detected in animals and mostly SDSD of bovine origin.

### 3.4 Novel *alp* family members can be found in different *Streptococcal* species

To investigate if the five novel *alp* family members and five mosaic variants were also present in other species, we performed protein BLAST of the N-terminal immunodomain and first repeat against the complete non-redundant NCBI protein database, with alignment and identity ≥ 90%. Homologous sequences were predominantly identified in SDSD/SDSE, but also in other *Streptococcal* species including GBS, GAS, *S. canis*, and *S. uberis* (**Table 2**). *Dysalp2* was detected most frequently, with 20 closely related homologs identified in GBS. In contrast, alp1–3hybrid, *dysalp3, dysalp4*, and *dysalp5* showed limited distribution, each with only one to four homologs identified exclusively in SD.

**Table 2:** Number of *Streptococcal* species hits detected by protein BLAST of N-terminal immunodomain and first repeat (as defined by predicted protein structure) against the complete non-redundant NCBI protein database, with alignment and sequence identity ≥ 90%.

Alp variant	Amino acids	Strain ID	GBS	*S. canis*	SD	SDSD	SDSE	GAS	*S. uberis*	Total
Alp1–3 hybrid	56-315	SDSE76					2			2
Dysalp2	56-311	iSDSE85		1				3		4
Dysalp2	56-311	iSDSE28	20		1				2	23
Dysalp3	56-314	iSDSE207			1					1
Dysalp4	56-315	SDSD35			1	3				4
Dysalp5	56-314	KUSD014					1			1
Total			20	1	3	3	3	3	2	35

### 3.5 *Alp* genes are present on MGEs in *S. dysgalactiae*

**Figure 4:**
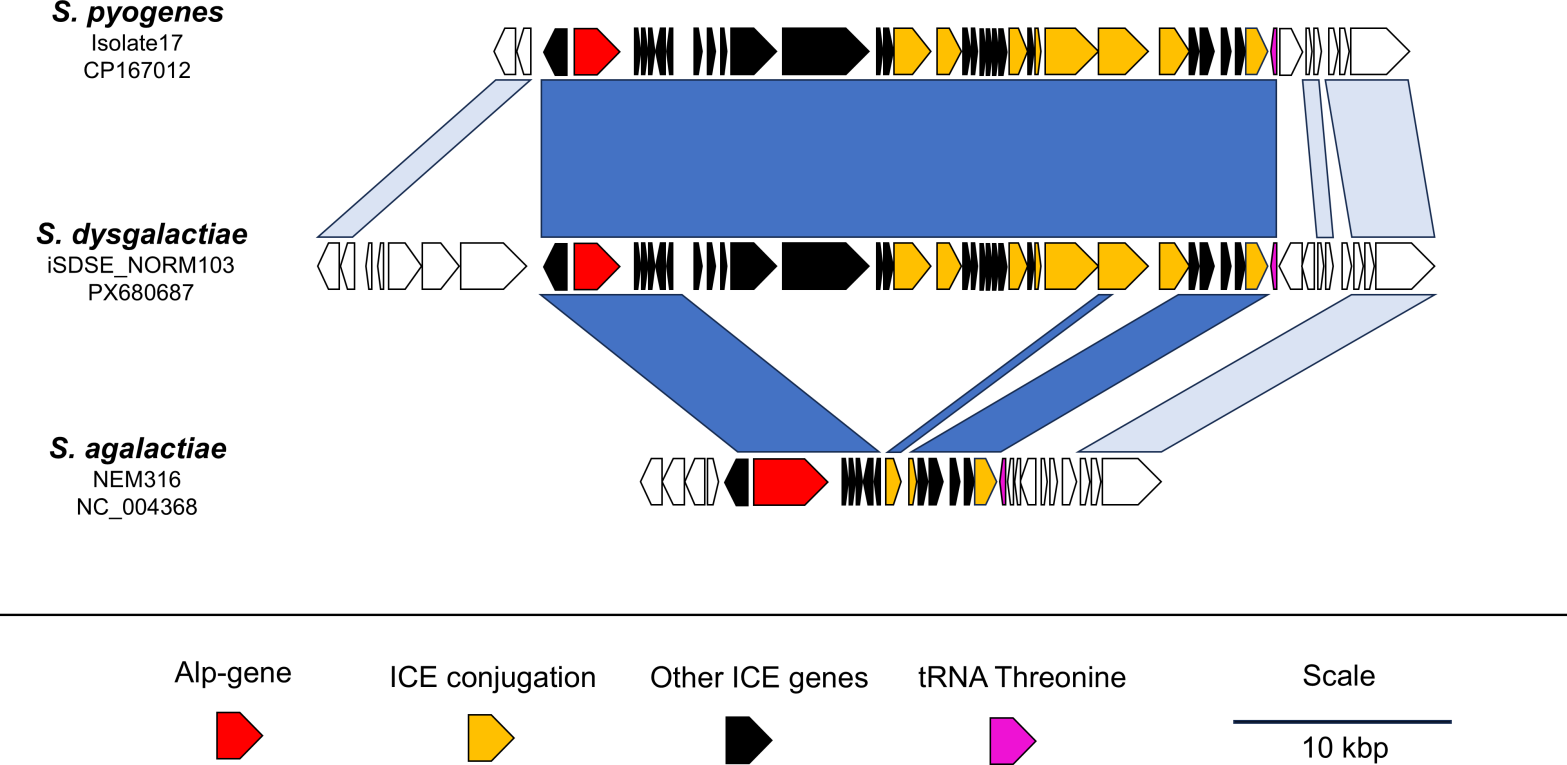
Comparative analysis of *alp3* and the flanking regions in *S. pyogenes*, *S. dysgalactiae* and *S. agalactiae*. The a*lp* genes are present on almost identical mobile genetic elements in *S. pyogenes* and *S. dysgalactiae*. Remnants of a similar element are detected also in many *S. agalactiae* isolates. Dark blue indicates > 99% nucleotide similarity, light blue indicates 85 – 90% homology.

Analyses of flanking sequences of the detected *alp* genes in SD strains of both human and animal sources revealed that these genes are located on MGEs belonging to the ICESt3 family. Comparing the elements to the ICE6180-RD2 element in GAS revealed > 99.9% similarity, differing by only six single nucleotide polymorphisms (**Figure 4**). A similar element was detected in all SD strains harboring *alp* genes. Varying degrees of homology among the conjugative genes were observed, though, inferring that the different *alp* genes were carried by related, but not identical, MGEs.

Interestingly, in GBS genomes deposited in GenBank, we found that *alp* genes were localized adjacent to conjugation genes similar to those in the ICE6180-RD2 element (**Figure 4B**). Nevertheless, we could not identify any GBS genomes harboring a complete set of conjugation genes. Most GBS genomes contained only the *alp* gene, an *alp* gene regulator, and an integrase, suggesting that these are decayed elements.

### 3.6 Protein structures of novel Alp family proteins

**Figure 5:**
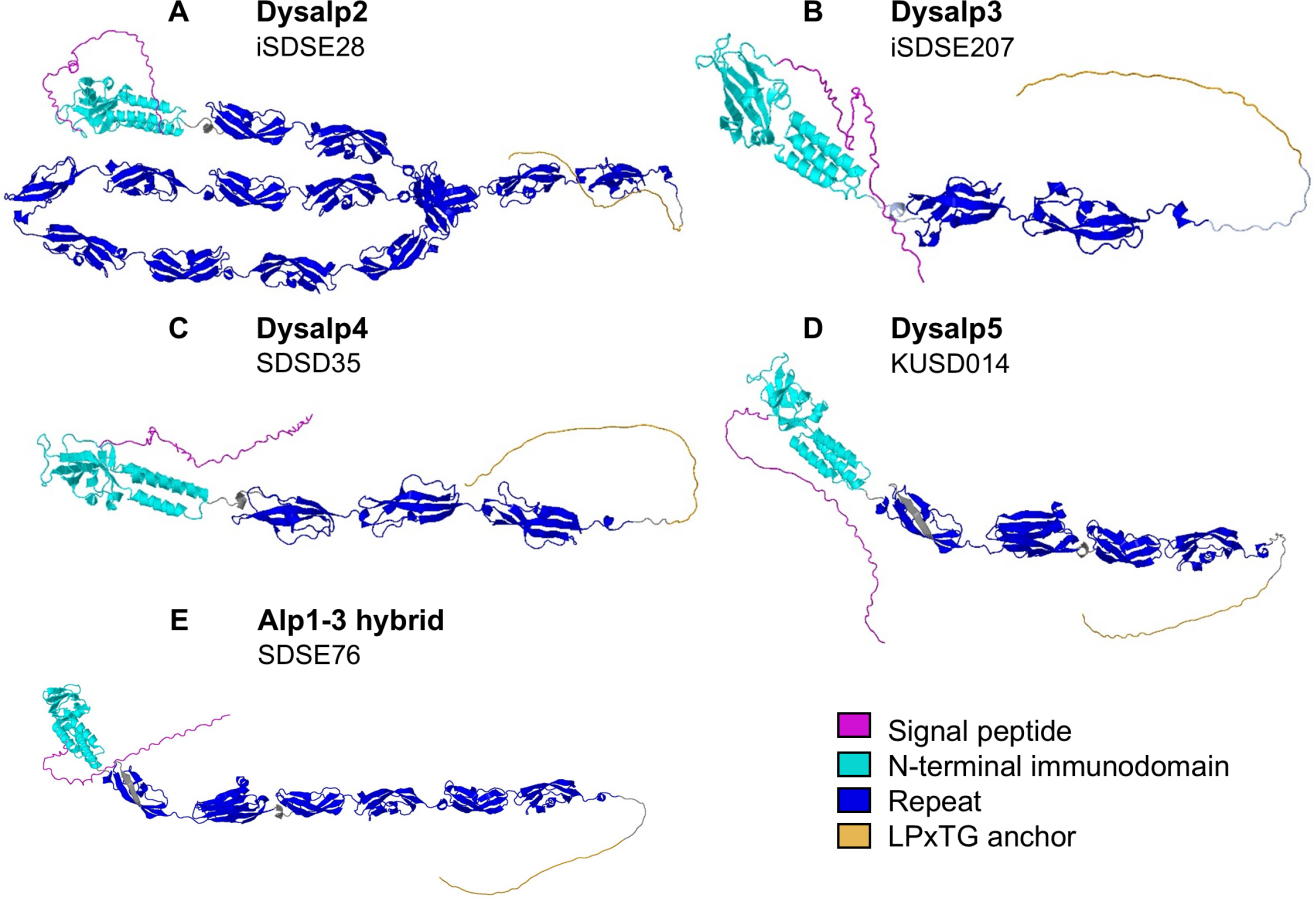
AlphaFold-predicted structures of novel Alps **(A-E)**. Structural domains indicated by colored key.

Next, we predicted the 3D structures of the novel Alp family proteins and mosaic variants using AlphaFold, yielding models with high overall confidence scores (**Figure 5**). The N-terminal immunodomain appeared to be conserved across Alp family proteins, comprising a β-sandwich and a three-helix bundle, consistent with the previously characterized Alpha C domain (Aupérin et al., 2005). The repeat regions also displayed a conserved core architecture resembling e.g. the Rib domain (Whelan et al., 2019), with each repeat forming a β-sandwich of antiparallel β-strands. Notably, the A and U domains shared the same structural architecture, possibly being derived from ancestral repeats that have undergone sequence divergence (**Supplementary Figure 1**). Repeat numbers varied from two to 14 and repeats were organized in a linear, beads-on-a-string arrangement (**Figure 5**). The unstructured interdomain linkers between repeats frequently contained proline, which suggests limited interdomain flexibility, as previously shown by Whelan et al. (2019) for Rib repeats.

## 4 Discussion

Alpha-like proteins are well characterized virulence determinants in both GBS and GAS, but their presence and role in other *Streptococcus* species is not well understood. To our knowledge, this is the first large in-depth genomic study exploring the occurrence of *alp* genes among SD strains. We describe five novel allelic variants of the Alp protein family genes and present the distribution of *alp* genes in SD from different geographical locations.

The novel allelic variants identified here exhibit the same mosaic pattern characteristic as *alp* genes previously described. For instance, the gene encoding Dysalp identified by Creti et al. (2007) comprises a combination of the Rib N-terminal immunodomain and Alp2 repeats. Similarly, *dysalp2* and *dysalp3* share repeat regions with *alp2* and *alp3*, respectively. *Dysalp4* is a mosaic encoding the N-terminal immunodomain of *alp2/3* combined with the repeat region of *alp1/bca*, while *dysalp5* represents a mosaic of *bca* and *alp3/rib*. Additionally, the N-terminal immunodomain of the Alp1–3 hybrid forms a chimera of the N-terminal immunodomains of Alp1 and Alp2/3, followed by Alp3-repeat regions.

Notably, we reveal that the diversity in *alp* genes extends far beyond the established constellations of immunodomains and repeat domains. There is a plethora of allelic combinations of A-domains, U-domains, and repeat types and quantity, challenging both precise identification and nomenclature. The mechanisms driving this hypervariability have yet to be elucidated, but tandem repeat variation and intra- and intergenomic recombination have been shown to be important modulators of surface-exposed virulence determinants (McNeilly and McMillan, 2014; Zhou et al., 2014).

We found *alp* genes to be common in SD, present in almost one fifth of our strains. Earlier studies have reported low prevalence of *alp* genes in SD. Gherardi et al. (2014) screened 54 SD strains from an Italian strain collection, including both carriage, invasive and non-invasive strains, for *alp* genes using PCR and detected them in only five strains (9%). However, because only PCR-based methods were employed, it is possible that novel *alp* variants were not detected, potentially underestimating the true prevalence and diversity of these genes in SD populations.

The prevalence and distribution of *alp* genes seemed to vary across strain collections. Notably, the Australian strains exhibited limited *alp* gene diversity with only *dysalp* detected. A similar limited *alp* gene diversity was evident in the Canadian strain collection, harboring exclusively *alp3*. In contrast, the strain collections from Norway and Japan had higher overall prevalence of *alp* genes and showed greater *alp* gene variation (**Table 1**). This is likely in part related to the studies’ different temporal and geographic boundaries. The Norwegian collection is based on nationwide sampling and a 20-year time-span, whereas strains from Australia were collected from two remote Aboriginal communities over a two-year period. Moreover, the Canadian, Japanese and Norwegian strains of human origin were all derived from invasive bacteremia, but the Australian strains were collected from throat swabs. Nevertheless, our findings infer temporal and geographic dynamics of circulating *alp* genes.

The *R28 alp3* homolog in GAS has been demonstrated to reside on the ICE6180-RD2 MGE (Sitkiewicz et al., 2011). In the present study, we find that all the *alp* genes in SD strains were localized on highly similar elements. This suggests a shared mechanism of mobility and integration between SD and GAS and supports the notion of active interspecies exchange. The finding of *alp* genes also in other *Streptococcal* species by BLAST search could indicate an even wider dissemination. Intriguingly, the *alp* genes are almost ubiquitous in GBS, but in line with previous reports, we find that their associated ICEs seem to have decayed (Sitkiewicz et al., 2011). A small proportion of clinical GBS strains lack an *alp* gene (Gabrielsen et al., 2017), suggesting that these genes may originally have been carried on MGEs that have since degraded or lost functionality. While direct evidence for active mobility of *alp* loci in GBS is lacking, the presence of ICEs and other MGEs in GBS genomes indicates that residual mobility or gene fragment exchange could still contribute to the distribution and diversity of *alp* genes (Brochet et al., 2008; Lorenzo-Diaz et al., 2016; Khan et al., 2023).

Alps are nearly ubiquitous in GBS, making this protein family an attractive target for vaccine development, particularly to prevent neonatal disease (Gabrielsen et al., 2017; McGee et al., 2021). A vaccine targeting the N-terminal immunodomain of GBS Alp-family proteins Rib, Bca, Alp1, and Alp2/3 (GBS-NN/NN2) is currently under clinical development (Banks et al., 2023). In the present study, 18% of human SD strains carry an *alp* gene, indicating that cross-protection against a subset of SD strains may be possible. The N-terminal immunodomains included in the GBS vaccine are also present in Dysalp4 and Dysalp5, as well as R28 in GAS, suggesting that vaccination could potentially provide protection against SD and GAS strains carrying these Alp variants. In contrast, potential protection against Dysalp2 and Dysalp3 is more uncertain. Of note, homologs of Dysalp2 were also detected in GBS, but appear to predominantly circulate among bovine strains, which may limit their relevance for human-targeted vaccines. Together, these findings highlight that Alp-based vaccines developed for GBS could offer partial cross-protection against other streptococcal species expressing related Alp proteins, although further studies are need to confirm this.

Most of the genomes analyzed in this study were generated using short-read sequencing, which presents challenges for accurate assembly. In three cases, the genomes were truncated to the extent that we could not assign a specific *alp* gene (**Table 1**). Additionally, this method does not allow for precise determination of repeat numbers. To address these limitations, we sequenced 32 strains from the Norwegian strain collections by Oxford Nanopore technology. As a result, we were able to define the complete domain architecture, determine the genomic context, and model the protein structures of novel Alp family allelic variants. Our structural analyses revealed a conserved core architecture that aligns with previously described domains, including the N-terminal immunodomain of Alpha C and the Rib repeat from *S. agalactiae* (Aupérin et al., 2005; Whelan et al., 2019). The Alp proteins exhibited a beads-on-a-string arrangement, with overall length dictated by the number of repeats. Our predictions furthermore indicated that the A- and U-domains were not discrete domains but rather components of domains sharing the common β-sandwich repeat structure. While Alp repeats have been reported to be highly conserved at the nucleotide level (Wästfelt et al., 1996), this structural similarity suggests a likely ancestral repeat origin, followed by sequence divergence and degeneration, yet with preservation of the fundamental domain structure. Thus, the conserved architecture observed in Alp proteins provides compelling evidence for shared functional roles across a broad spectrum of mosaic variants and species.

In conclusion, alpha-like proteins are common and widely distributed among SD. They exhibit extensive genetic heterogeneity, predominantly arising from mosaicism and tandem repeat variations, and we identified five novel *alp* members. The *alp* genes in SD are carried by mobile genetic elements, and the detection of highly similar elements across streptococcal species suggests potential for dissemination.

## Data Availability

The datasets presented in this study can be found in online repositories. The names of the repository/repositories and accession number(s) can be found in the article/**Supplementary Material**.
