# Supplementary material for: The dynamic Alp landscape of Streptococcus dysgalactiae
Source: Front Cell Infect Microbiol. 2026 Apr 10;16:1770747. doi: 10.3389/fcimb.2026.1770747 (PMC13106166; doi:10.3389/fcimb.2026.1770747)
Supplement: Supplementary file 1 [file DataSheet1.pdf]

Supplementary Material

Supplementary Figure 1

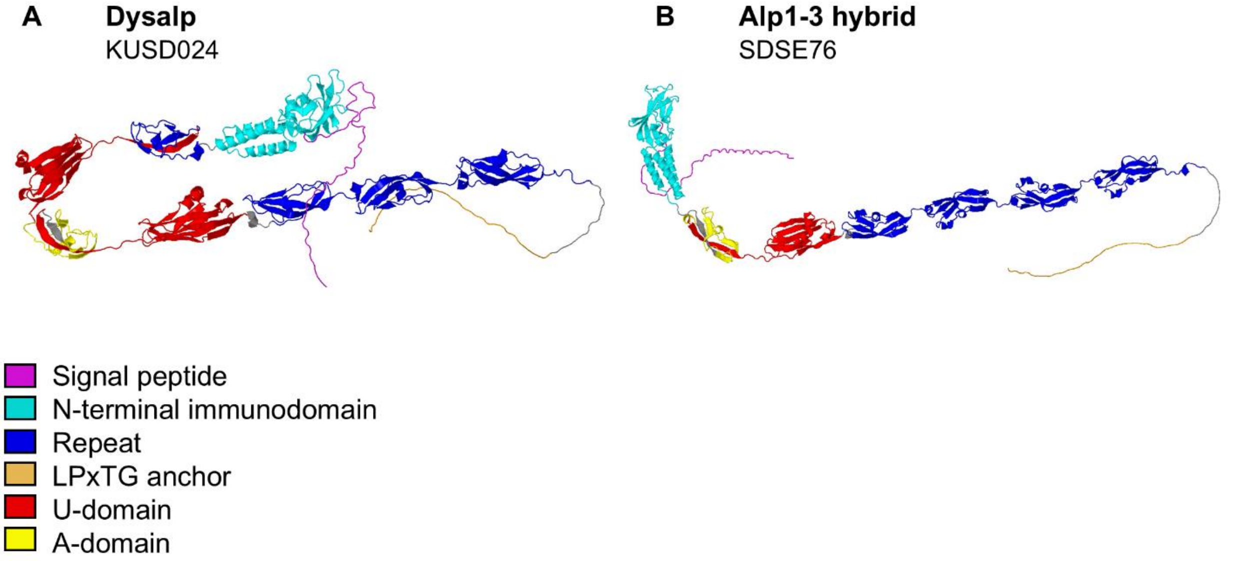

Supplementary Figure 1. A and U domains share the same structural architecture as the repeat domains as seen in Dysalp-strain KUSD024 (A) and the Alp1-3 hybid-strain SDSE76 (B). Structural domains are indicated by colored key.
